# Reply To: Relative tree cover does not indicate a lagged Holocene forest response to monsoon rainfall

**DOI:** 10.1038/s41467-022-33959-6

**Published:** 2022-10-24

**Authors:** Jun Cheng, Haibin Wu, Zhengyu Liu

**Affiliations:** 1 School of Marine Sciences, Nanjing University of Information Science and Technology, Nanjing, 210044 China; 2 Key Laboratory of Cenozoic Geology and Environment, Institute of Geology and Geophysics, Chinese Academy of Sciences, Beijing, 100029 China; 3 CAS Center for Excellence in Life and Paleoenvironment, Beijing, 100044 China; 4 University of Chinese Academy of Sciences, Beijing, 100049 China; 5 Department of Geography, Ohio State University, Columbus, OH 43210 USA

**Keywords:** Palaeoecology, Palaeoclimate

**replying to** Y. Cheng et al. *Nature Communications* 10.1038/s41467-022-33958-7 (2022)

We welcome the comment from Matters arising from Cheng Y et al.^1^, which provides us an opportunity for further clarification of some of our points^2^. The Comment raised interesting and important issues about our paper, that undoubtably could enhance our understanding for the Holocene vegetation evolution in the northern China and its relationship with East Asian Summer Monsoon (EASM). In particular, the results from Dali lake pose the questions on the timing of the peak of tree cover, that invokes the further investigation to understand this complex tree changes over Holocene period. However, these comments do not impact the key result in our original study^2^, that is the periodical asynchronous evolutions between EASM and northern China ecosystem under specific conditions.

Main points of our paper are: First, we propose that the EASM and its rainfall over northern China mainly followed the variation of the summer insolation and peaked in the early Holocene, while the relative tree cover of **temperate deciduous broadleaf tree** peaked in the mid-Holocene; the delayed tree cover peak is caused by the winter warming, and peak soil moisture also in the mid-Holocene, which could be related to a hydrological impact from vegetation shift from grass to tree and the positive feedback between this vegetation shift and soil wetting.

Second, this asynchronous evolution between the EASM rainfall, which peaks in the early Holocene, and the northern China ecosystem, which peaks in the mid-Holocene, is caused mainly by the opposing effect of residual ice sheet retreat on the decreasing summer insolation. The declining summer insolation does cause a substantial decrease of EASM rainfall from the early to mid-Holocene. However, 2/3 of this rainfall decrease is canceled by the rainfall increase forced by the retreat of residual Laurentide ice sheet, resulting in a weak decreasing trend of rainfall over this period.

Third, under this background of weak rainfall changes, winter warming, induced by increased winter insolation and ice sheet retreat, raised the coldest temperature to above −17 °C, the threshold for the survival of **temperate deciduous broadleaf tree**^3^, and then favored an increase in tree, meanwhile induced a decrease in grass for reasons of its lower competitiveness than that of tree. This vegetation shift then supported the wetting of northern China through its hydrological effect^2^. The vegetation shift and soil wetting could reinforce each other. Furthermore, the dominant effect of winter warming on vegetation from the early to mid-Holocene is supported by our sensitive experiments with an off-line land-vegetation model.

As stated in Cheng Y’s Comment^1^, the land cover in northern China includes forests, grass and bare land. In our interpretation, the process of “the vegetation feedback to climate” is mentioned as a possible feedback that enhances this asynchronous response, but is not critically involved in the mechanism. As such, whether the absolute or relative vegetation cover is not a major issue in our discussion. It’s sure that the reconstructed absolute tree cover, which based on pollen concentration, could enrich our understanding of the vegetation changes over the Holocene period in northern China. Indeed, the hydrological impact of bare land (evaporation) had been considered in our hydrological analysis of northern China soil moisture, and the results indicated its impact is important but not critical to the Holocene long-term changes of soil moisture over this region. The relative tree cover, the percentage of cool mixed tree (COMX^4^) in fossil pollen which is consistent with that of **temperate deciduous broadleaf tree** in simulation^2^, that we cited^4^ is a synthesis of 31 records, which represented the general evolution of vegetation over a large part of northern China, and its main result is consistent with records from other part of northern China such as the 6 ka peak in Gonghai Lake^5^. In spite of its low time resolution, the general trend over the millennium scale seems to us clear.

It’s true that the −17 °C of the coldest month temperature is the survival threshold for the temperate deciduous broadleaved tree. While, the temperature threshold for C_3_ grass and C_3_ arctic grass are complex, its direct impact on the changes of grass proposed in our paper is somewhat not strict. However, considering the different competitiveness between tree and grass, increased temperate deciduous broadleaved tree, which derived by the winter warming, could induce a decrease in the grass from the early to mid-Holocene. Indeed, summer temperature, annual rainfall and fire incidents are all the important factors determining the Holocene changes of vegetation over northern China, but series of sensitivity experiment proposed the key impact of winter temperature on the vegetation shift and soil moisture evolution, which is consistent with the results of transient coupled climate simulation and geological records. This grass-to-tree shift for this period is evidenced in the pollen percentages and well simulated by the climate model shown in our paper^2^.

Fire is an important factor for the long-term changes of vegetation cover over semi-arid regions, and its emergence and impact on vegetation are already incorporated into our model^6^, then, in turn, the simulation. Future works could assess the impact of fire on the long-term changes of semi-arid vegetation through the combination of reconstruction and process-based simulation of fire^7^.

Focusing on the contrary views of Holocene EASM within proxy records, we proposed an asynchronous evolution of EASM rainfall and northern China ecosystem for the period of early to mid-Holocene. Our proposal is based on a state-of-the-art transient climate simulation, which reproduced the diverse evolution of EASM proxies reasonably well. The mechanisms proposed for this asynchronous evolution appear to us consistent with the current evidences available. There are, however, uncertainties in models and proxies. Meanwhile, the northern China is a broad region with large gradient in rainfall and ecosystem, that could induce the possible diverse evolutions in climate and ecosystem under Holocene climate change. Therefore, we believe further studies using other models and new proxies are important to further improve our understanding of this issue.

## Data Availability

No new data were generated for this reply.
